# Frailty, delirium and hospital mortality of older adults admitted to intensive care: the Delirium (Deli) in ICU study

**DOI:** 10.1186/s13054-020-03318-2

**Published:** 2020-10-15

**Authors:** David Sanchez, Kathleen Brennan, Masar Al Sayfe, Sharon-Ann Shunker, Tony Bogdanoski, Sonja Hedges, Yu Chin Hou, Joan Lynch, Leanne Hunt, Evan Alexandrou, Manoj Saxena, Simon Abel, Ramanathan Lakshmanan, Deepak Bhonagiri, Michael J. Parr, Anders Aneman, Danielle Ni Chroinin, Kenneth M. Hillman, Steven A. Frost

**Affiliations:** 1 Critical Care Research in Collaboration and Evidence Translation, Penrith, Australia; 2 Department of Intensive Care, Campbelltown Hospital, Campbelltown, Australia; 3 Department of Intensive Care, Bankstown-Lidcombe Hospital, Bankstown, Australia; 4 Department of Intensive Care, Fairfield Hospital, Fairfield, Australia; 5 Department of Intensive Care, Liverpool Hospital, Liverpool, Australia; 6 Western Sydney University, Penrith, Australia; 7 South Western Sydney Clinical School, University of New South Wales, Kensington, Australia; 8 Department of Geriatric Medicine, Liverpool Hospital, Liverpool, Australia; 9 Simpson Centre for Health Services Research, Ingham Institute of Applied Medical Research, Liverpool, Australia; 10 South Western Sydney LHD Centre for Applied Nursing Research, Ingham Institute of Applied Medical Research, Liverpool, Australia

**Keywords:** Frailty, Delirium, ICU outcomes

## Abstract

**Background:**

Clinical frailty among older adults admitted to intensive care has been proposed as an important determinant of patient outcomes. Among this group of patients, an acute episode of delirium is also common, but its relationship to frailty and increased risk of mortality has not been extensively explored. Therefore, the aim of this study was to explore the relationship between clinical frailty, delirium and hospital mortality of older adults admitted to intensive care.

**Methods:**

This study is part of a Delirium in Intensive Care (Deli) Study. During the initial 6-month baseline period, clinical frailty status on admission to intensive care, among adults aged 50 years or more; acute episodes of delirium; and the outcomes of intensive care and hospital stay were explored.

**Results:**

During the 6-month baseline period, 997 patients, aged 50 years or more, were included in this study. The average age was 71 years (IQR, 63–79); 55% were male (*n* = 537). Among these patients, 39.2% (95% CI 36.1–42.3%, *n* = 396) had a Clinical Frailty Score (CFS) of 5 or more, and 13.0% (*n* = 127) had at least one acute episode of delirium. Frail patients were at greater risk of an episode of delirium (17% versus 10%, adjusted rate ratio (_adj_RR) = 1.71, 95% confidence interval (CI) 1.20–2.43, *p* = 0.003), had a longer hospital stay (2.6 days, 95% CI 1–7 days, *p* = 0.009) and had a higher risk of hospital mortality (19% versus 7%, _adj_RR = 2.54, 95% CI 1.72–3.75, *p* < 0.001), when compared to non-frail patients. Patients who were frail and experienced an acute episode of delirium in the intensive care had a 35% rate of hospital mortality versus 10% among non-frail patients who also experienced delirium in the ICU.

**Conclusion:**

Frailty and delirium significantly increase the risk of hospital mortality. Therefore, it is important to identify patients who are frail and institute measures to reduce the risk of adverse events in the ICU such as delirium and, importantly, to discuss these issues in an open and empathetic way with the patient and their families.

## Introduction

The population is ageing worldwide; the 841 million people older than 60 years in 2013 is estimated to more than double to 2 billion by 2050 [23]. This increase in life expectancy has been influential in changing the characteristics of older patients admitted to the intensive care unit (ICU) and has highlighted frailty as an important emerging clinical problem [1, 2, 4–6]. Among older patients admitted to the ICU, an acute episode of delirium is also common, and has been suggested as a sign of brain frailty [10, 13, 16, 21], and is associated with a longer intensive care and hospital stay [8, 17], and increased risk of mortality. The complex relationship between frailty, delirium and risk of mortality has not been extensively explored in the ICU setting. Therefore, the aim of this study was to explore the relationship between clinical frailty on admission, an episode of delirium and hospital mortality of older adults admitted to the ICU.

## Methods

This study of the relationship between clinical frailty, delirium and hospital mortality is part of a larger, nurse-led interventional study, to reduce the burden of delirium in the adult ICU setting, which has been described previously [15]. In brief, the Delirium in ICU (Deli) Study is a randomised stepped-wedge intervention trial, including the four adult intensive care units across the South Western Sydney Local Health District (SWSLHD). The intervention is a nurse-led non-pharmacological bundle of care, to reduce the incidence of delirium among adults admitted to the ICU. The data for this specific study of the relationship between frailty, delirium and hospital mortality is based on the baseline period (pre-intervention phase) of the larger *Deli Study*. This sub-study was planned prior to the commencement of data collection on 1 May 2019.

### Subjects and setting

The South Western Sydney Local Health District (SWSLHD) provides public hospital services for around a million residents, with five acute care hospitals, with approximately 230,000 separations each year. There are four adult ICUs (one tertiary referral and three metropolitan), with between 80 and 250 admissions each per month.

### Ethical considerations

This project was considered by the South Western Sydney Local Health District Human Research Ethics Committee and was determined to meet the requirements of the National Statement on Ethical Conduct in Human Research (2007). Due to the nursing intervention being implemented among all admissions, and the use of routinely collected ICU and hospital separation data, the need for individual patient consent was waived (HREC ref.: HE18/169; Australian New Zealand Clinical Trials Registry (ANZCTR) (ref no. ACTRN12618000411246p)).

### Inclusion and exclusion criteria

Consecutive patients admitted during the study period were enrolled in the study, excluding patients with delirium on admission, those not expected to stay in the ICU very long and any patient that we would not be able to assess for delirium. This includes (1) patients at the end-of-life and not expected to survive 24 h; (2) patients not expected to stay in the ICU for at least 24 h; (3) patients with acute or chronic neurological conditions that may prevent assessment of delirium (traumatic brain injury, intra-cerebral haemorrhage, ischaemic stroke, central nervous system infection, hypoxic brain injury, hepatic encephalopathy, severe mental disability, serious receptive aphasia, severe dementia); and (4) patients with persistent coma, preventing the assessment of delirium.

### Data collection

Specific data collected for the study included age, sex, admission date and discharge from ICU and hospital, ICU and hospital outcomes, and clinical frailty status on admission to intensive care, along with identification of an acute episode of delirium. Other general characteristics of the patients on admission to ICU were collected from the Hospital Health Information Exchange (HIE) and the Australian and New Zealand Adult ICU data collection. History of comorbid conditions was obtained using ICD-10-AM codes; a Charlson Index was calculated, using the method suggested by Quan et al. [18].

### Assessment of clinical frailty

Clinical frailty status was assessed on admission to the ICU by the admitting medical officer, either directly from the patient, their family or review of any previous medical notes. Frailty was collected using Rockwood’s Clinical Frailty Score [19]. Frailty status was based on the patient’s level of physical function in the 2 months prior to their admission to the hospital for the index ICU stay during the study period. Admissions with a Clinical Frailty Score (CFS) of 5 or more were classified as frail [2, 19].

### Identification of delirium

The *Confusion Assessment Method* (CAM) was used to identify acute episodes of delirium among any patient who appears to be disorientated or confused, or who has any change in behaviour or level of consciousness [12] during an ICU stay. The CAM is based on four main areas of assessment: (1) *acute onset and fluctuating course* (is there evidence of an acute change in mental status from baseline? If so, did the abnormal behaviour fluctuate during the day?), (2) *inattention* (did the patient have difficulty focussing attention during the interview?), (3) *disorganised thinking* (was the patient’s thinking disorganised?) and (4) *altered level of consciousness* (overall, how would you rate the patient’s level of consciousness?) [12]. Patients who were rousable (Richmond Agitation and Sedation Scale ≥ − 3) were assessed for the presence of delirium using the CAM [12] or CAM-ICU [7]. Both versions have been validated as a reliable (kappa = 0.96; 95% CI 0.91–0.99) and valid (sensitivity 0.81–0.82 and specificity 0.99) tool for diagnosing delirium in the ICU setting [7, 22]. Our hospital-based electronic medical record system (eMR) currently only offers the CAM for documentation. However, all ICU staff are trained to use both the CAM and the CAM-ICU (for example, when a patient is unable to verbalise, the *inattention* and *disorganised thinking* components of the CAM are assessed using the CAM-ICU approach. We have a single standard delirium policy and protocol that is used across our four adult ICUs. We did not perform any specific reliability assessments of the CAM and CAM-ICU during the study period.

Delirium status was assessed each shift by nursing and/or medical staff (shifts range from 8 to 12 h in duration) or when there was an acute change in mental status. On each morning of admission during an ICU stay (up to a maximum of 21 days), patients were recorded as delirium yes, if at least one episode was recognised by clinical staff during the last 24-h period, or delirium free. Each recorded delirium event was further categorised to be of a hypoactive, hyperactive or mixed nature [7].

### Outcomes of interest

The outcomes of interest for our analysis were (1) clinical frailty status on admission to ICU, (2) rates of acute episodes of delirium in the ICU, (3) rates of ICU mortality, (4) length of stay in the ICU and hospital and (5) hospital mortality.

### Sample size

The sample size planned for the overall baseline and intervention phase of the Deli Study was based on monthly admissions between 80 and 125 (adults, aged 16 years or more) patients from the four ICUs included in the 12-month study [15]. Our local health district ICU data estimated approximately 80% of admissions were among patients aged 50 years or more and that after application of the inclusion and exclusion criteria, approximately 70% of admissions would be included in our study. Using the baseline (6 months) period of the Deli Study, we estimated approximately 1008 patients (aged 50 years or more) would be included in our analysis of the relationship between frailty, delirium and hospital mortality. A post hoc power calculation based on a 15% rate of hospital mortality among non-frail patients, and a 33% rate of frailty [2], our estimated sample size of 1008 for the baseline period would have a power of 0.79 to detect a 50% increase in the risk of hospital mortality among frail patients compared to non-frail patients.

### Statistical analysis

Characteristics of patients admitted to the four adult ICUs during the baseline 6-month period of the Deli Study are presented using descriptive statistics. Risk of hospital mortality was based on at least one episode of delirium in the ICU during the study period and frailty status. Crude and adjusted rate ratios (RR) and 95% confidence intervals (95% CI) were estimated using a generalised linear model (Poisson error) [3]. Due to the potential complex relationship between frailty, delirium and subsequent risk of mortality, the role of delirium being an effect modifier of the risk of death due to frailty was assessed by including an interaction term between frailty and delirium, and hospital death. A *p* value of < 0.1 was used to confirm the interaction between frailty and delirium, and then crude and adjusted (adjusted for age and sex) models were estimated for delirium-free and delirium patients [3]. Data imputation was not planned for missing data. All data management and analyses were performed using the R statistical language (R Core Team, 2018) [20].

## Results

**Fig. 1:**
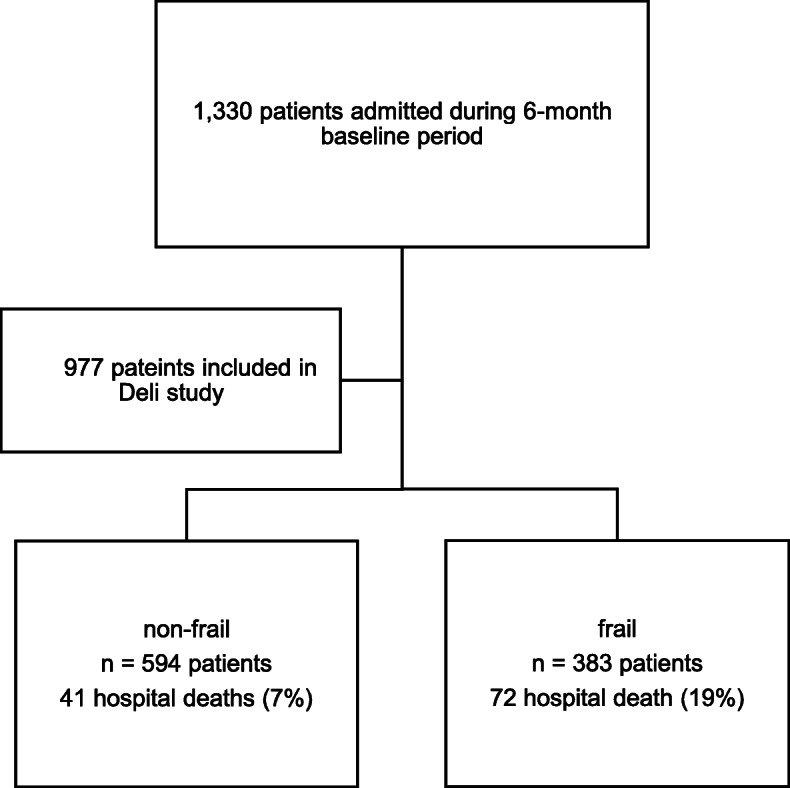
Flow diagram of participants included in the study, frailty status and hospital mortality

The total numbers of admissions to the four adult ICUs during the baseline period, and the number of patients included in the study aged 50 years or more, once inclusion and exclusion criteria were applied are presented in Fig. 1. Due to the stepped-wedge nature of the overall 12-month Deli Study, each ICU individually crossed over from baseline to interventions after the initial 3-month baseline period; the four ICUs contributed 3 to 6 months of data for analysis. Based on clinical frailty status on admission to ICU, the characteristics and outcomes of the 997 patients included in this study are presented in Table 1. For example, 39.2% (95% CI 36.1–42.3%, *n* = 396) had a Clinical Frailty Score (CFS) of 5 or more on admission to ICU; frail patients were older (77 versus 67 years, *p* < 0.001), were more likely female (50% versus 42%, *p* = 0.021) and had a higher rate of multiple admissions to ICU during the study period (6% versus 4%, *p* = 0.017). Delirium was more common among the frail (17% versus 10%, *p* = 0.002) compared to the non-frail; length of stay in the ICU and hospital were longer, 3 versus 2 days for ICU stay (*p* < 0.001) and 11 versus 9 days for hospital stay (*p* < 0.001), respectively. And frail patients had higher rates of ICU and hospital mortality, 10% versus 3% for ICU death (*p* < 0.001) and 19% versus 7% for hospital death (*p* < 0.001). Based on the age group, frailty status, rates of delirium, ICU and hospital length of stay, and mortality are presented in Table 2. Older patients had higher rates of frailty, delirium and mortality and stayed in the ICU and hospital longer (all *p* values for trend < 0.01).

**Table 1:** Characteristics of admissions, aged 50 years or more, to intensive care during the baseline study period, based on frailty status

Clinical Frailty Score of 5 or more on admission to intensive care				
	Yes (*n* = 383)	No (*n* = 594)	Combined (*n* = 977)	*p* value
Age (years), median (IQR)	77 (69–83)	67 (60–75)	71 (63–79)	< 0.001
Males, *n* (%)	193 (50)	344 (58)	537 (55)	0.021
Single admission to ICU, *n* (%)	361 (94)	572 (96)	933 (95)	0.017
Delirium, *n* (%)	66 (17)	61 (10)	127 (13)	0.002
Charlson Index, median (IQR)	2 (1–3)	1 (0–2)	1 (1–3)	< 0.001
No. of comorbidities, median (IQR)	2 (1–3)	1 (1–2)	1 (1–2)	< 0.001
Dementia, *n* (%)	15 (4)	3 (1)	18 (2)	< 0.001
Admitted from ED, *n* (%)	172 (45)	218 (37)	390 (40)	0.011
Planned surgery, *n* (%)	22 (6)	84 (14)	106 (11)	< 0.001
APACHE III, median (IQR)	64 (51–81)	50 (38–66)	56 (42–71)	< 0.001
Mechanical ventilation, *n* (%)	53 (14)	97 (16)	150 (15)	0.292
ICU death, *n* (%)	39 (10)	17 (3)	56 (6)	< 0.001
Hospital death, *n* (%)	72 (19)	41 (7)	113 (12)	< 0.001
ICU LOS (days), median (IQR)	3 (2–5)	2 (1–4)	2 (1–5)	0.001
Hospital LOS (days), median (IQR)	11 (6–24)	9 (5–16)	10 (5–19)	< 0.001

Characteristics of ICU stay are from the first admission to ICU during the study period

**Table 2:** Characteristics of admissions, to intensive care during the baseline study period, based on age group

	Age group (years)			
	50–64 (*n* = 298)	65–79 (*n* = 443)	80+ (*n* = 236)	*p* value^a^
Frail, *n* (%)	62 (21)	164 (37)	157 (67)	< 0.001
Delirium, *n* (%)	26 (9)	56 (13)	45 (19)	< 0.001
Males, *n* (%)	168 (56)	344 (58)	537 (55)	0.021
ICU LOS (days), median (IQR)	2 (1–4)	3 (2–5)	2 (1–4)	0.284
ICU death, *n* (%)	9 (3)	24 (5)	43 (18)	0.001
Hospital death, *n* (%)	72 (19)	24 (7)	113 (12)	< 0.001
Hospital LOS (days), median (IQR)	8 (4–15)	10 (6–20)	11 (6–24)	0.004

^a^*p* value for trend

Based on the frailty status, risks of delirium and hospital mortality are presented in Table 3. Frail patients were at greater risk of an episode of delirium (17% versus 10%, adjusted rate ratio (_adj_RR) = 1.71, 95% confidence interval (CI) 1.20–2.43, *p* = 0.003), and a higher risk of hospital mortality (19% versus 7%, _adj_RR = 2.54, 95% CI 1.72–3.75, *p* < 0.001), when compared to non-frail patients. Frail patients, not being recognised to experience an episode of delirium during and ICU stay, had a 15.5% rates of hospital mortality, compared to a rate of 6.6% among non-frail patients without delirium (_adj_RR = 2.24, 95% CI 1.37 to 3.67, *p* = 0.001). Patients who were frail on admission to ICU and experienced an acute episode of delirium in the ICU had a 35.9% rate of hospital mortality, versus 9.9% among non-frail admissions who also experienced delirium in the ICU (_adj_RR = 4.16, 95% CI 1.50 to 11.52, *p* = 0.004).

**Table 3:** Frailty, delirium and risk of hospital mortality

	**Risk of delirium in ICU**			*p* value^1^
	Crude rate ratio (95% CI)		Adjusted rate ratio (95% CI)	
Frailty (17%) versus non-frail (10%)	1.68 (1.18–2.38)		1.71 (1.20–2.43)	0.003
Age (each 10-year increase)	1.02 (0.99–1.06)		1.01 (0.96–1.06)	0.711
Males (14.7%) versus females (10.3%)	1.39 (0.97–2.00)		1.45 (1.01–2.08)	0.042
	**Risk of hospital death**			
	Crude rate ratio (95% CI)		Adjusted rate ratio (95% CI)	
Frail (19%) versus non-frail (7%)	2.72 (1.86–4.00)		2.54 (1.72–3.75)	< 0.001
Delirium (23%) versus non-delirium (10%)	2.31 (1.51–3.52)		2.03 (1.33–3.12)	0.002
Age (each 10-year increase)	1.02 (0.98–1.06)		1.01 (0.95–1.07)	0.875
Males (11%) versus females (12%)	0.93 (0.64–1.34)		0.94 (0.65–1.37)	0.753
	**Effect modification of delirium on the risk of hospital death due to frailty**			
	Deaths/total (%)	Crude rate ratio (95% CI)	Adjusted rate ratio (95% CI)	
Non-frail (non-delirium)	35/533 (6.6%)	1.0 (ref)	1.0 (ref)	
Frail (non-delirium)	49/317 (15.5%)	2.60 (1.64–4.11)	2.24 (1.37–3.67)	0.001
Non-frail (delirium)	6/61 (9.9%)	1.0 (ref)	1.0 (ref)	
Frail (delirium)	23/66 (35.9%)	4.90 (1.83–13.1)	4.16 (1.50–11.52)	0.004

^1^Adjusted for age and sex

## Discussion

**Fig. 2:**
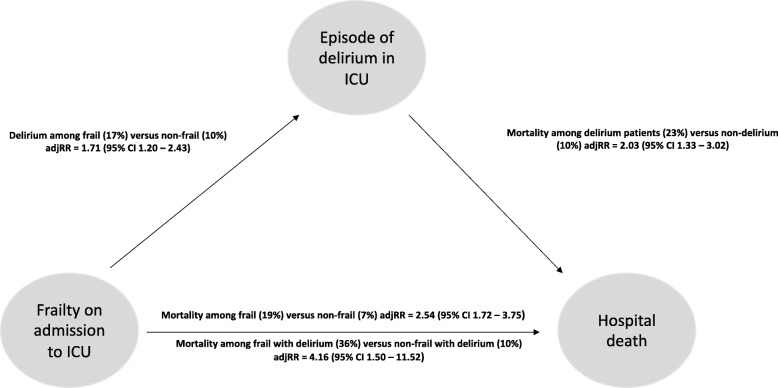
Hospital mortality based on frailty and delirium status. _adj_RR, adjusted for age, sex and rate ratio

This study among adults, aged 50 years or more, admitted to the ICU has been able to show that clinical frailty on admission increases the risk of delirium, resulted in a longer ICU and hospital stay, and increases the risk of in-hospital mortality. Importantly, our study suggests that a proportion of the effect of frailty on the increased risk of hospital mortality is modified by delirium, and one in three frail patients who experienced an acute episode of delirium in the ICU did not survive to hospital discharge (Fig. 2). These results suggest the importance of recognising clinical frailty in the ICU setting, not just to improve the prediction of outcomes from critical illness, but to identify patients at the greatest risk of adverse events such as delirium; to institute measures to reduce risk; and hopefully improve outcomes. The presence of frailty on admission to the ICU may also be considered as a marker of someone nearing the end of their life. This prognostic information together with inherent uncertainty should be shared with the patient and their families in an honest and empathetic way.

The prevalence of clinical frailty among adults admitted to the ICU has been previously described [2, 5, 9] and, along with the results of our study, showed longer lengths of stay and increased risk of mortality. However, our study may be one of the first to specifically explore the relationship between frailty, delirium and risk of death. The prevalence of frailty among adults aged 50 years or more was reported to be 32.8% (95% CI 28.3–37.5%), in Alberta, Canada [2], from six hospitals, and is similar to our rate of 39.2% (95% CI 36.1–42.3%). Frailty among the very elderly (aged 80+ years) admitted to the ICU has also been extensively explored, in terms of ICU and hospital outcomes [5, 9, 11], and the increased prevalence in this very elderly group has highlighted frailty as an important predictor of short-term mortality. Importantly, this study has described the moderating effect of delirium in the relationship between frailty and increased risk of hospital mortality, in that frail patients who experience an episode of delirium in the ICU are at the greatest risk hospital death.

The results of our study need to be considered in the context of some potential weaknesses and strengths. Firstly, the classification of frailty in the clinical setting, especially among critically ill patients, is difficult—however, the work by Rockwood et al. in developing the Clinical Frailty Score (CFS) has made this task easier. For instance, the CFS used in this study has demonstrated a similar concordance to the more detailed cumulative deficit method in predicting 28-day mortality, among study participants in the Canadian Health and Ageing Cohort [19]. And given the impracticality of using a comprehensive geriatric assessment in the ICU setting to identify frailty, the risk of our study participants being misclassified is a potential weakness.

Another obvious problem is the identification of delirium in the ICU setting is often subject to some error. However, the majority of this error is related to false negatives (sensitivity of 0.81) when the CAM and CAM-ICU had been compared to a more exhaustive assessment of delirium using the Diagnostic and Statistical Manual of Mental Disorders (DSM IV) [22]. Importantly, false-positive rates have been estimated to be low (1%) when also compared to DSM IV [22]. The consequences of this would be that delirium rates may be underestimated, and using the method suggested by Kelsey et al. [14] (using the above estimates of sensitivity and specificity of 0.81 and 0.99, respectively), the overall observed rate of 12.5% would increase to approximately 14.6%. Also, our inclusion and exclusion criteria may account for our lower rates of delirium, which was based on our larger (12 months) interventional study and may limit the generalisability of our results to all patients admitted to the ICU. A strength of this study is that it has been conducted across a number of adult ICUs and that both frailty and delirium statuses were purposely collected as part of our larger study.

The implications of our finding are twofold: (1) frailty in the ICU setting is common and needs to be routinely identified as part of the characteristics of patients admitted to the ICU and (2) frail patients in the ICU are at greater risk of adverse events, such as delirium, and have worse hospital and long-term outcomes. Further work in this area needs to identify modifiable risk factors to reduce the risk of adverse events, such as delirium, among this vulnerable group of patients cared for in the ICU, and explore more extensive outcomes of those who are frail and survive an ICU and hospital stay, for instance, functional outcomes (both at discharge and at 6- and 12-month follow-up), quality of life and longer-term mortality in the months following discharge from hospital.

## Conclusion

This study among adults, aged 50 years or more, admitted to the ICU has been able to show that clinical frailty on admission increases the risk of delirium by 60%. The effect of frailty on the increased risk of hospital mortality is moderated by delirium, and one in three frail patients who experience an acute episode of delirium during their stay in the ICU did not survive to hospital discharge. These results suggest the importance of recognising clinical frailty in the ICU setting, not just to improve the prediction of outcomes from critical illness, but to identify patients at the greatest risk of adverse events such as delirium, and institute measures to reduce risk, improve health outcomes where possible and share prognostic information in a genuine way with the patient and their carer.

## Data Availability

Reasonable request for access to data and material can be organised through the last author.
